# Health-Related Quality of Life (HRQoL) of Residents with Persistent Lower Respiratory Symptoms or Asthma Following a Sulphur Stockpile Fire Incident

**DOI:** 10.3390/ijerph19052915

**Published:** 2022-03-02

**Authors:** Shahieda Adams, Mayuri Rajani, Roslynn Baatjies, Faieza Omar, Mohamed Fareed Jeebhay

**Affiliations:** 1 Occupational Medicine Division and Centre for Environmental and Occupational Health Research, School of Public Health and Family Medicine, University of Cape Town, Observatory 7925, South Africa; shahieda.adams@uct.ac.za (S.A.); mayuriraj@hotmail.co.uk (M.R.); roslynn.baatjies@uct.ac.za (R.B.); desai.tariq@gmail.com (F.O.); 2 Department of Environmental and Occupational Studies, Faculty of Applied Sciences, Cape Peninsula University of Technology (CPUT), Cape Town 7535, South Africa

**Keywords:** persistent respiratory symptoms, asthma, health-related quality of life, upper airways, lower airways

## Abstract

*Background:* This study evaluated health-related quality of life (HRQoL) in residents with persistent lower respiratory symptoms (PLRS) or asthma six years after exposure to sulphur dioxide vapours emanating from an ignited sulphur stockpile. *Methods:* A cross-sectional study was carried out, using interview data collected at three time points (prior to, one- and six-years post incident), medical history, respiratory symptoms and HRQOL using the Medical Outcomes Study Form 36 (SF-36). *Results*: A total of 246 records, 74 with and 172 without PLRS or asthma, were analysed. The mean age was 42 (SD:12) years in the symptomatic group and 41 (SD:13) years in the asymptomatic group. Mean SF-36 scores were significantly lower for the symptomatic group in the Physical Functioning (24 vs. 39), Role—Physical (33 vs. 48) and General Health (GH) domains (24 vs. 37). Symptomatic residents experienced a significant decline in their Role—Physical (OR = 1.97; CI 1.09, 3.55) and GH (OR = 3.50; CI 1.39, 8.79) at year 6 compared to asymptomatic participants. Residents with co-morbid reactive upper airways dysfunction syndrome demonstrated stronger associations for GH (OR = 7.04; CI 1.61, 30.7) at year 1 and at year 6 (OR = 8.58; CI 1.10, 65.02). *Conclusions:* This study highlights the long-term adverse impact on HRQoL among residents with PLRS or asthma following a sulphur stockpile fire disaster.

## 1. Introduction

Exposure to sulphur dioxide following air pollution and industrial incidents has been associated with the development of poor respiratory health and adverse outcomes in exposed individuals [1,2,3]. A few studies have reported long-term respiratory health effects post incident among individuals exposed to high levels of sulphur dioxide, manifesting mainly as bronchial hyperresponsiveness, persistent lower respiratory symptoms (PLRS), irritant-induced asthma and chronic obstructive pulmonary disease (COPD) [4,5]. 

PLRS following incidental exposure to high levels of chemicals has also been reported in workers cleaning up after an oil spill up to five years following exposure [6]. Workers and communities exposed to chemicals following the World Trade Centre explosion in New York also demonstrated persistent respiratory symptoms during follow-up surveillance medical examinations [7,8,9,10]. Individuals affected by such exposures and resultant respiratory morbidity may experience reduced health health-related quality of life (HrQoL) [11]. Various studies have investigated the impact of asthma on HRQoL using well-validated instruments [12,13,14] and have consistently documented impaired physical health [12,13,14,15]. Furthermore, strong positive associations have been observed between severity of asthma and HRQoL.

Other studies have also reported reduced HRQoL and adverse mental health impacts in rescue/recovery workers for years following the World Trade Centre disaster [8,9,10,16]. Such effects have ranged from post-traumatic stress symptoms to probable mental health disorder (PTSD, depression or general anxiety disorder) and reduced HrQoL in those with PLRS. A study evaluating the relationship between exposure, adverse health impacts and HrQoL in first responders of the WTC incident demonstrated that chronic health conditions, such as rhinosinusitis and asthma, affected HrQoL more than a decade after the incident. The study also demonstrated that years later WTC exposures did not independently affect HrQoL but instead influenced HrQoL.

We previously reported PLRS asthma in Macassar residents following a large sulphur stockpile fire incident at a chemical manufacturing plant six years after the incident [1]. 

At the time of the disaster, the population of the nearby Macassar township was exposed as a consequence of almost half of the sulphur stockpiled on the premises (7250 tons) being consumed by the fire. This resulted in 14,500 tons of SO_2_, the primary combustion product, being released into the environment. The fire raged on for about 21 h, with varying levels of intensity, producing SO_2_ concentrations ranging between 3–55 ppm, with the highest exposures (>100 ppm) being experienced in the first 7 h due to the strong prevailing north-westerly wind. Over 4000 residents from the area had to be evacuated at the time, with many of these residents experiencing various acute symptoms attributable to the fire. Upper and lower respiratory symptoms (LRS) were the most common symptoms reported within the first week after the fire [1].

These PLRSs were the result of aggravation of pre-existing asthma or new onset acute irritant induced asthma (commonly referred to as reactive airways dysfunction syndrome—RADS). It is this subgroup of individuals that is the subject of the current study aimed at determining HRQoL in residents with and without PLRS or asthma, six years after exposure to sulphur dioxide vapours emanating from the fire disaster.

## 2. Materials and Methods

### 2.1. Recruitment and Study Sample

**Figure 1:**
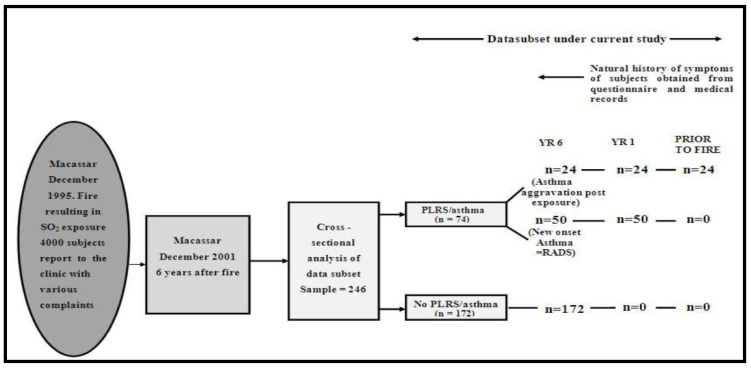
Sample of participants affected by the sulphur stockpile fire incident included in the study.

A cross-sectional analysis of data collected over a period of 24 months was undertaken. The study population dataset originally consisted of 4000 residents of Macassar who presented six years later to the Macassar disaster project clinic for a medical evaluation following health complaints as a result of acute exposure to sulphur dioxide vapours at the time of the fire incident. Within this group, there were residents with pre-existing asthma that experienced further aggravation of their asthma and a proportion of residents that developed a new onset of PLRS or asthma (Figure 1). Study participants had to meet certain criteria. All participants were Macassar residents at the time of the fire incident and had to be eighteen years or older at the time of the incident. All participants underwent a medical evaluation at the Macassar disaster project clinic and had to be free of PLRS, asthma and other chronic respiratory illness, such as pneumonia or chronic obstructive pulmonary disease, at the time of the disaster. In addition, none had pulmonary TB (PTB) at least one year prior to the disaster. Individuals with PLRS included those that reported persistent lower respiratory symptoms (LRS)/asthma at year 1 (Yr1) and year 6 (Yr6) after the incident, which in the opinion of the medical panel (MRP) was probably related to inhaling SO_2_. Participants in the comparison group included those who were randomly selected from the larger study population database and identified to be free of PLRS or asthma following the incident following the routine medical examinations [1].

Sample size was calculated using STATA version 10 statistical software (StataCorp, College Station, TX, USA). This was computed based on a background exposure prevalence of 25% and 50% among residents with PLRS or asthma and residents without PLRS or asthma, respectively, providing 80% power and 95% confidence. All residents with PLRS or asthma were selected and a random sample of residents without PLRS or asthma were selected. Based on these calculations, a sample of 74 adult residents with PLRS or asthma and 172 adult residents without PLRS or asthma were selected for the analysis. All residents participating in the study at the time of the medical examination provided written consent and the research protocol was approved by the Research Ethics Committee of the University of Cape Town (REC REF: 474/2009).

### 2.2. Questionnaires

Information from residents was obtained through a structured interviewer-administered questionnaire. The first part of the questionnaire was used to obtain data pertaining to demographic characteristics, exposure history, co-morbidities and health symptoms experienced prior to, at year 1 and 6 years after the incident. Additional information on socio-economic status and health care utilisation was not collected from the participants since these variables were not considered to be differentially distributed among the residents of this low socio-economic residential area. The second part of the questionnaire collected data on HRQoL that consisted of the generic health status questionnaire of the SF-36 health survey [17,18]. The SF-36 questionnaire incorporates eight scales, viz. Physical Functioning (PF), Role—Physical (RP), Bodily Pain (BP), General Health (GH), Role—Emotional (RE), Mental Health (MH), Vitality (VT) and Social Functioning (SF). 

### 2.3. Definition of PLRS or Asthma

Persistent lower respiratory symptoms included wheezing, chest tightness or a doctor’s diagnosis of obstructive lung disease (irritant induced asthma, asthma aggravation or COPD), present at year 1 and 6 years after the incident. Participants were asked about respiratory symptoms that were present before, 2 weeks after, within 1 month and 1 year after the incident. At follow-up they were asked whether they had experienced symptoms in the past 4 weeks. A panel of at least three experienced occupational medicine specialists reviewed the information obtained from the questionnaire and medical records provided by the individual’s personal doctor and the clinical examination to assess the presence of PLRS, their relationship to the incident and the need for further investigations. These diagnoses were then confirmed by a specialist pulmonologist who obtained an independent detailed medical history, conducted a clinical examination and other appropriate investigations (chest radiographs and lung function tests). The pulmonologist provided a final diagnosis for each individual after completing the clinical evaluation.

### 2.4. Statistical Analysis

Statistical analysis was performed using STATA version 10 (StataCorp, College Station, TX, USA). The Rand 36-item scoring procedure was used. A two-step scoring process was implemented; the first step involved recoding some items, and step 2 involved averaging the items in the same scale. The scores from all the eight scales ranged from 0 to 100, with a high score being consistent with positive health status [17].

The eight scales of the SF-36 HRQoL were classified as the dependant variables with the main predictor of interest being PLRS or asthma. The other covariates considered were age, sex/gender, smoking, current treatment of asthma, previous history of pulmonary TB, hypertension, cardiac disease, diabetes, arthritis, depression and anxiety. Normality of data was explored using the Shapiro–Wilk test. The non-parametric Mann–Whitney test was used to test for differences in the scores of the eight SF-36 scales between residents with and without PLRS or asthma, 6 years after the fire. The mean differences for seven of the SF-36 scales (missing data for scale Physical Functioning prior to the fire and year 1) were calculated by subtracting the SF-36 HRQoL scale scores for year 1 from the baseline scores and similarly, for year 6, stratified according to the presence and absence of PLRS or asthma. 

Stepwise multiple linear regression analysis (with the eight SF-36 scales as the dependent variables) was performed to determine the relationship between the eight SF-36 scales scores and the presence of the predictor of interest and the other covariates of age, sex/gender, smoking status, pulmonary TB, reactive upper airways dysfunction syndrome (RUDS), hypertension, cardiac disease, diabetes, arthritis, depression and anxiety. Multivariate logistic regression models adjusted for age, sex/gender, smoking, pulmonary TB, cardiac history and depression (anxiety was not included in the model as it was found to be highly correlated with depression) were used to determine the contribution of PLRS or asthma towards decline in HRQoL with as well as without co-existing RUDS morbidity. A decline of more than five points in the SF-36 (0–100 scale) was considered to be of clinical significance [19].

## 3. Results

The demographic characteristics of the entire study population are presented in Table 1. Overall, the mean age of residents was 42 (SD: 13) years, with almost 60% being current or ex-smokers and a greater proportion being females (61.3%). While these characteristics were generally comparable between residents with PLRS or asthma and those without PLRS or asthma, a previous history of pulmonary TB was more prevalent in the former group. A large proportion of residents (72.7%) reported suffering from one or more chronic diseases. The prevalence of depression and anxiety was similar, while cardiac disease and diabetes were more common in residents with PLRS and/or asthma, with arthritis more prevalent in residents without PLRS or asthma (Table 1).

In the analysis of those with symptoms, a significant proportion (69%) of residents who reported PLRS or asthma were women. Among the 74 residents with PLRS or asthma, 63% reported a doctor’s diagnosis of reactive airways dysfunction syndrome (RADS) and 32% asthma aggravation, while 42% had reactive upper airways dysfunction syndrome (RUDS). All the residents with RUDS had co-existing RADS. A high proportion (83%) of residents with asthma had impaired lung function, with 37% classified as mild (FEV_1_: 61–80% predicted), 18% moderate (FEV_1_: 51–60% predicted) and 28% with severe (FEV_1_ < 50% predicted) impairment (data not shown). Notably, only 60% of these residents were on current asthma treatment at 6 years follow-up.

The overall mean scores for each of the SF-36 scales at year 6 are presented in Table 2, viz. Physical Functioning 29 ± 27.3, Role—Physical 44 ± 48.3, Bodily Pain 64 ± 30.9, General Health 33 ± 24.8, Vitality 51 ± 20.6, Social Functioning 60 ± 28.6, Role—Emotional 49 ± 49.3 and Mental Health 51 ± 20.6. At year 6, residents with PLRS or asthma scored lower for all domains of the SF-36 scale except for Bodily Pain, compared to those without PLRS or asthma. A similar pattern was observed for the scores at year 1 (Supplementary Table S1 online). The scores representing the physical health component scales, viz. Physical Functioning, Role—Physical and General Health, were significantly lower in residents with PLRS or asthma (*p* < 0.05). Furthermore, the mean differences in scores were significantly lower for Role—Physical (−36 vs. −24; *p* = 0.036) and General Health (−50 vs. −30; *p* < 0.001) at year 1 and for General Health (−50 vs. −38; *p* = 0.004) at year 6 in residents with PLRS or asthma (Supplementary Table S2 online).

Multivariate linear regression models demonstrated that all statistically significant (*p* < 0.05) predictors of HRQoL investigated were inversely correlated with the SF-36 scale scores (Table 3 and Table 4). Furthermore, in addition to PLRS or asthma, age, sex/gender, depression, anxiety and cardiac disease were the strongest determinants of physical HRQoL scores, while age, sex/gender, smoking status, depression and anxiety were important predictors of mental HRQoL scores. Aside from depression and anxiety, residents with PLRS or asthma were more likely (*p* < 0.05) to have lower scores for the following three SF-36 scales: Physical Functioning (−13.13), Role—Physical (−13.09) and General Health (−12.94) at year 6. The Physical Functioning model explained the greatest variability in the HRQoL scores (33%). 

A significantly larger proportion of residents with PLRS or asthma reported clinically significant decline (five points) in scores for the SF-36 scales compared to the asymptomatic group at year 1, viz. Role—Physical (40.5% vs. 27.3% *p* = 0.04) and General Health (92% vs. 62% *p* < 0.001). Similarly, a significantly higher proportion of residents with PLRS or asthma experienced a significant decline in their General Health compared to residents without PLRS or asthma (91.8% vs. 76.7% *p* = 0.005), at year 6 (data not shown). 

Logistic regression models for investigating the determinants of decline of more than five points in the SF-36 health scale demonstrated a decline in for Role—Physical (OR = 1.97; CI 1.09, 3.55) and General Health (OR = 7.07; CI 2.88, 17.35) at year 1 and General Health (OR = 3.50; CI 1.39, 8.79) at year 6 in residents with PLRS or asthma (Table 5). Similarly, residents with PLRS or asthma with co-existing RUDS experienced a clinically significant decline (more than five points) in their HRQoL for General Health (OR = 7.04; CI 1.61, 30.7) at year 1 and at year 6 (OR = 8.58; CI 1.10, 65.02). The model with RUDS on its own yielded similar results to the combined model that included PLRS or asthma, since RUDS always co-existed with PLRS or asthma (data not shown). 

## 4. Discussion

This study has shown that residents who developed PLRS or asthma as a result of exposure to SO_2_ vapours during an environmental disaster experienced a negative impact on their HrQoL. This is reflected in significantly lower scores (*p* < 0.05) for the SF-36 scales Physical Functioning, Role—Physical and General Health compared to residents without PLRS or asthma 6 years after the incident. PLRS or asthma were strong determinants of physical as opposed to mental HRQoL. Furthermore, residents with co-morbid RUDS also scored significantly lower on the SF-36 subscale General Health.

Residents with PLRS or asthma reported lower General Health perception, as they perceived their health to be poorer and believed that it would get worse over time. This group also had lower scores in Physical Functioning and Role—Physical indicating that they had experienced limitations in performing physical activities and daily activities of living or work at 6 years after the incident. Furthermore, residents with PLRS or asthma were more likely to experience decline (more than five points from baseline) in their HRQoL for the SF 36 scales Role—Physical (OR = 1.97) and General Health (OR = 7.07) after 1 year and General Health (OR = 3.50) 6 years after the incident. This is in keeping with a study that demonstrated a mediating role for chronic health conditions, such as rhinosinusitis and asthma, PTSD and depression linked to chemical exposures, affecting HRQoL more than a decade after the exposure [11].

In this study, residents with PLRS or asthma experienced significant impairment in physical health up to 6 years after the fire incident. This is reflected in the high proportion of moderate to severe asthmatics (46%) relative to mild asthmatics (37%) in this group. Previous studies have also demonstrated significantly greater impairment in HRQoL in physical health in severe to moderate asthmatics compared to mild asthmatics [12,13,14,15]. Muraki et al. reported that patients with severe asthma experienced poorer HRQoL as measured on the SF-36 scale of Physical Functioning [15]. In population-based studies of asthmatics, instruments used to measure HrQoL, such as the AQLQ questionnaire and to a lesser extent the generic SF-36 questionnaire, have been shown to be sensitive to asthma severity.

In keeping with a negative influence on physical performance, those residents with severe asthma were also shown to experience a negative impact on HRQoL, work productivity and activity [20]. Song et al. similarly showed that asthma severity, being female, of an older age and having a lower level of education were significantly associated with lower physical component scores in a large HRQoL survey [21]. The role of asthma control in modifying asthma severity and resultant quality of life is an important factor that needs to be considered, which was not taken into account in this study [22]. Contrary to our findings, Osman et al.’s study of mild asthmatics reported both physical and mental HRQoL effects [23]. In a large population-based study, however, mental health scores and wellbeing appeared to be less affected by asthma severity, as has been shown in our study [21].

It is argued that individuals with chronic diseases, such as asthma, employ coping mechanisms to deal with their asthma by reducing their expectations for their heath and activities. Thus, it may well be the case that residents with moderate to severe PLRS or asthma may have adapted to the physical restrictions caused by their asthma, resulting in an increased sense of subjective wellbeing and better quality of life [24]. This could contribute to a more positive outlook on life and less mental HRQoL effects, as reported by studies of mild asthmatics.

In our study, PLRS and/or asthma and persistent RUDS (irritant-induced rhinitis) occurred concomitantly in the chronic phase, indicating that they were highly correlated. Notably, no one had RUDS without RADS, pointing to the unified airway model of pathogenesis. Furthermore, those with RUDS and PLRS reported poorer General Health perceptions at year 6 and over time. Leynaert et al. found that patients with allergic rhinitis experienced greater impairment in their mental health, with significantly lower scores for the SF-36 scales for General Health and Vitality and for the Mental Component Summary (MCS) [25]. However, patients with both rhinitis and asthma experienced greater impairment in their physical health, particularly in the SF-36 domains of Physical Functioning and Role—Physical. Hence, asthma was found to further impair HRQoL in patients with both rhinitis and asthma. Kalpaklioglu et al. also reported that patients with asthma experienced significant impairment in their physical health. Moreover, in patients with both allergic rhinitis and asthma there appeared to no further impairment of HRQoL [26].

The association of female sex with poorer HRQoL is borne out by other studies that have consistently demonstrated a reduced quality of life score in females when compared to male asthmatics [27,28,29]. This disparity remained even when baseline health status and levels of asthma control similar to males were achieved. Females reported a worse perception of their asthma, experienced more symptoms and experienced a greater impact on their HRQoL [29,30]. Several explanations for gender differences in the association between rhinitis or asthma and HRQoL have been proposed, the first being related to the severity of illness, with women commonly suffering from more severe rhinitis or asthma compared to men. Secondly, female sex hormones are also hypothesized to affect these outcomes, with a lower incidence of asthma linked to menopause and a higher incidence linked to puberty and hormonal therapy [19]. However, other studies have reported female patients to experience worse HRQoL despite having the same severity of asthma as their male counterparts [27,31]. Therefore, gender differences may also be due to differences in response to symptoms, with women being more distressed by their asthma symptoms whereas men may not report on the impact on their HRQoL until their symptoms become severe and long-standing [32]. Differences in adherence to medication (females less inclined to use inhaled medication) and differences in management of female patients (females less likely to undergo spirometry) have also been postulated to contribute to these gender disparities in HRQoL linked to asthma [29].

A strong association between co-morbid mental health conditions (anxiety and depression) and lowered HRQoL scores for both physical and mental health parameters was shown in this study. Notably, studies in respondents and communities affected by the World Trade Centre explosions have focussed on the persistence of lower respiratory symptoms (LRS), PTSD and HRQoL [8,9,10]. Participants with PLRS were three times more likely to report poor physical health and approximately 50% more likely to report poor mental health than the no LRS group [9]. Responders who had evidence of both LRS and PTSD were approximately three times more likely to report only fair or poor general health and more than twice as likely to report being unable to perform usual activities. This highlights the need to prioritize surveillance and mental and physical healthcare for survivors of complex environmental disasters.

A strength of this study is that it used a well-validated instrument, the SF-36 health survey, which enabled the assessment of both mental and physical parameters of health, among participants. Furthermore, this was an opportunistic study of a rare event. The Macassar sulphur fire disaster was a complex environmental polluting incident and provided an opportunity to assess the impact of co-existing PLRS or asthma and RUDS on HRQoL in residents exposed to SO_2_ on such a large scale [1].

Being an opportunistic study, the results are subject to several limitations. Firstly, ‘self-selection’ bias, since the entire study population was self-referred and not randomly selected and may have been driven by secondary gain factors, such as financial compensation. Secondly, recall bias may have occurred, since residents were interviewed six years after the fire incident. Recall bias cannot be discounted since secondary gain factors may have been at play. Furthermore, the reporting would not have been differentially distributed among residents with PLRS or asthma and those that were asymptomatic. Finally, the study was potentially subject to confounding, given that data for socio-economic status (education, occupation and income) and health care utilisation were not collected. However, these variables were not considered to be differentially distributed amongst the two groups and were therefore not considered to have significantly impacted on the findings. Data on important factors, such as treatment adherence and access to care, were also not collected and these factors may have had a modifying influence on HRQoL.

The findings of this study highlight the public health impact of an incidental high level of sulphur dioxide environmental exposure on affected residents. Health care providers need to be cognisant of the perceived impairment in physical health in residents with PLRS or asthma and general quality of life following such exposures. Consideration needs to be given to increased awareness of enduring physical limitations experienced by these residents long after exposure has ceased and the influence these adverse health impacts have on HRQoL. Ongoing access to health services, focussing on both mental and physical health, should be provided to such affected communities to monitor the adverse impacts on respiratory health and HRQoL through post-incident medical surveillance.

## 5. Conclusions

In conclusion, this study demonstrates a long-term adverse impact on HRQoL among residents reporting upper and lower airway disease following a sulphur stockpile fire disaster. It highlights the need for long-term surveillance and care of communities affected by such exposures to optimise their health and mitigate the effects these incidents may have on their quality of life.

## Figures and Tables

**Table 1:** Demographic and health-related characteristics of Macassar residents stratified according to the presence of persistent lower respiratory symptoms and/or asthma 6 years after the fire incident (*n* = 246).

Demographic Characteristics	Total N (%)	PLRS or (*n* = 246) Symptoms after the Fire (*n* = 74) N (%)	No PLRS or Symptoms after the Fire (*n* = 172) N (%)	*p*-Value
* Age (years) Mean ± SD	42 ± 12.8	42 ± 11.8	41 ± 13.2	0.494
Gender				
Female	162 (65.8)	52 (70.3)	110 (63.9)	
Male	84 (34.2)	22 (29.7)	62 (36.1)	0.338
Smoking status				
Never	101 (41.1)	30 (40.5)	71 (41.2)	
Previous	81 (32.9)	29 (39.1)	52 (30.2)	
Current	64 (26.0)	15 (20.2)	49 (28.4)	0.271
Previous Pulmonary Tuberculosis	29 (11.8)	13 (17.5)	16 (19.3)	0.065
Current treatment for asthma	45 (18.3)	45 (60.8)	0 (0)	<0.001
Other chronic diseases				
Hypertension	61 (24.8)	17 (22.9)	44 (25.5)	0.664
Cardiac disease	19 (7.72)	8 (10.8)	11 (6.3)	0.234
Diabetes	22 (8.94)	10 (13.5)	12 (6.9)	0.099
Arthritis	25 (10.2)	3 (4.0)	22 (12.7)	0.038
Depression	23 (9.3)	8 (10.8)	15 (8.7)	0.606
Anxiety	29 (11.8)	10 (13.5)	19 (11.0)	0.582

Chi-squared tests were used to analyse differences for all variables between groups. * For age, a *t*-test was used to analyse and compare groups.

**Table 2:** Health-related quality of life SF-36 scale scores of Macassar residents stratified according to the presence of persistent lower respiratory symptoms or asthma 6 years after the fire incident (*n* = 246).

	Physical Functioning Score		Role—Physical Score		Bodily Pain Score		General Health Score	
	Mean (SD)	Median (IQR)	Mean (SD)	Median (IQR)	Mean (SD)	Median (IQR)	Mean (SD)	Median (IQR)
PLRS/Asthma	24 ± 25.1	15 (0–35)	33 ± 45.9	0 (0–100)	67 ± 30.8	78 (33–100)	24 ± 17.8	19 (13–32)
No PLRS/Asthma	39 ± 28.5	35 (18–35)	48 ± 48.7	0 (0–100)	67 ± 31.0	78 (33–100)	37 ± 25.9	32 (13–56)
*p*-value *	<0.001		0.024		0.797		<0.001	
	**Vitality Score**		**Social Functioning Score**		**Role—Emotional Score**		**Mental Health Score**	
	**Mean (SD)**	**Median (IQR)**	**Mean (SD)**	**Media (IQR)**	**Mean (SD)**	**Median (IQR)**	**Mean (SD)**	**Median (IQR)**
PLRS/Asthma	50 ± 20.1	50 (35–65)	57 ± 28.8	55 (33–80)	46 ± 49.2	0 (0–100)	62 ± 26.9	60 (36–88)
No PLRS/Asthma	52 ± 20.6	50 (40–65)	62 ± 28.2	68 (43–80)	51 ± 49.1	63 (0–100)	63 ± 24.9	60 (44–84)
*p*-value *	0.685		0.239		0.535		0.883	

* Statistical test of significance: Mann–Whitney test.

**Table 3:** Significant determinants of health-related quality of life according to SF-36 subscale scores for physical health domains of Macassar residents (*n* = 246) 6 years after the fire incident using multivariate linear regression models.

	β-Coefficient (95% Confidence Interval)			
	PF	RP	BP	GH
Constant	89.18 (77.12, 101.25) ***	85.35 (62.58, 108.11) ***	91.08 (76, 105.57) ***	44.59 (39.27, 49.90) ***
Age (years)	−0.78 (−1.02, −0.55) ***	−0.70 (−1.169, −0.27) **	−0.45 (−0.74, −0.16) **	-
Female	−19.09 (−25.30, −12.89) ***	-	-	−9.98 (−16.16, −3.80) **
Smoking				
- Previous	-	-	-	-
- Current	-	-	-	-
Previous Pulmonary TB	-	-	-	-
PLRS/asthma	−13.13 (−19.56, −6.71) ***	−13.09 (−25.86, −0.34) *	-	−12.94 (19.19, −6.49) ***
RUDS	-	-	-	-
Depression	-	−26.32 (−46.39, −6.26) **	-	−10.10 (−21.01, −0.89) *
Anxiety	-	-	−21.50 (−33.06, −9.93) ***	-
Cardiac disease	−10.72 (−22.11,0.66) ^δ^	-	-	-
Model R^2^	0.33	0.08	0.08	0.06

Scales: PF—Physical Functioning, RP—Role—Physical, BP—Bodily Pain, GH—General Health. RUDS—Reactive Upper Airways Dysfunction Syndrome; *: *p* < 0.05; **: *p* < 0.01; ***: *p* < 0.001, ^δ^ *p* = 0.5–0.65.

**Table 4:** Significant determinants of health-related quality of life according to SF-36 subscale scores for mental health domains of Macassar residents (*n* = 246) 6 years after the fire incident using multivariate linear regression models.

	β-Coefficient (95% Confidence Interval)			
	V	SF	RE	MH
Constant	64.40 (54.78, 74.01) ***	62.63 (59.00, 66.26) ***	98.89 (74.58, 123.20) *	77.42 (71.08, 83.77) ***
Age (years)	−0.23 (−0.42, −0.03) *	-	−0.75 (−1.22, −0.28) **	-
Female	-	-	−14.39 (−27.09, −1.70) *	−9.57 (−15.77, −3.37) **
Smoking	-	-	-	−4.84 (−8.49, −1.19) **
- Previous	-	-	-	−6.82 (−13.61, −0.04) *
- Current	-	-	-	−9.80 (−17.05, −2.54) **
Previous Pulmonary TB	-	-	-	-
PLRS/asthma	-	-	-	-
RUDS	-	-	-	-
Depression	−13.98 (−22.81, −5.15) **	−25.24 (−37.11, −13.37) ***	−21.85 (−42.45, −1.25) *	−19.64 (−29.82, 9.26) ***
Anxiety	−7.38 (−15.35, 0.58) ^δ^	-	-	−17.32 (−26.76, −7.88) ***
Cardiac disease	-	-	-	-
Model R^2^	0.08	0.06	0.09	0.2

Scales: V—Vitality, SF—Social Functioning, RE—Role—Emotional, MH—Mental Health. RUDS—Reactive Upper Airways Dysfunction Syndrome; *: *p* < 0.05; **: *p* < 0.01; ***: *p* < 0.001, ^δ^ *p* = 0.5–0.65.

**Table 5:** Determinants of decline in health-related quality of life in physical health according to SF-36 subscale scores of Macassar residents (*n* = 246) at 1 year and 6 years after the fire incident using multivariate logistic regression models.

	Decline in SF-36 Scores after 1 Year Odds Ratio (95% CI)			Decline in SF-36 Scores after 6 Years Odds Ratio (95% CI)		
	Role—Physical	Bodily Pain	General Health	Role—Physical	Bodily Pain	General Health
PLRS/asthma	1.97 (1.09, 3.55) *	1.24 (0.70, 2.21)	7.07 (2.88, 17.35) ***	1.59 (0.89, 2.84)	1.05 (0.59, 1.84)	3.50 (1.39. 8.79) **
PLRS/asthma and RUDS	1.82 (0.84, 4.15)	0.95 (0.42, 2.13)	7.04 (1.61, 30.7) **	1.65 (0.74, 3.79)	0.84 (0.38, 1.84)	8.58 (1.10, 65.02) *

Each odds ratio is a separate model adjusted for age, sex, smoking status, pulmonary TB, depression and cardiac disease. *: *p* < 0.05; **: *p* < 0.01; ***: *p* < 0.001. RUDS—Reactive Upper Airways Dysfunction Syndrome.

## Data Availability

Data are provided in the online Supplementary Materials and the corresponding author may be contacted if any clarification is needed regarding the data used.

## Supplementary Materials

The following supporting information can be downloaded at: https://www.mdpi.com/article/10.3390/ijerph19052915/s1, Table S1: Health-related quality of life SF-36 scale scores of Macassar residents prior to and at 1 year and 6 years after the fire (*n* = 246); Table S2: Mean difference in the health-related quality of life SF-36 scale scores of Macassar residents stratified according to the presence of persistent lower respiratory symptoms and/or asthma 6 years after the fire (*n* = 246).

## References

[1] Baatjies R., Adams S., Cairncross E., Omar F., Jeebhay M.F. (2019). Factors associated with persistent lower respiratory symptoms or asthma among residents exposed to a sulphur stockpile fire incident. Int. J. Environ. Res. Public Health.

[2] Chen R., Huang W., Wong C.M., Wang Z., Thach T.Q., Chen B., Kan H., CAPES Collaborative Group (2012). Short-term exposure to sulfur dioxide and daily mortality in 17 Chinese cities: The China air pollution and health effects study (CAPES). Environ. Res..

[3] Chen T.M., Kuschner W.G., Gokhale J., Shofer S. (2007). Outdoor air pollution: Nitrogen dioxide, sulfur dioxide, and carbon monoxide health effects. Am. J. Med. Sci..

[4] Piirilä P.L., Nordman H., Korhonen O.S., Winblad I. (1996). A thirteen-year follow-up of respiratory effects of acute exposure to sulfur dioxide. Scand. J. Work. Environ. Health.

[5] Andersson E., Knutsson A., Hagberg S., Nilsson T., Karlsson B., Alfredsson L., Torén K. (2006). Incidence of asthma among workers exposed to sulphur dioxide and other irritant gases. Eur. Respir. J..

[6] Zock J.P., Rodríguez-Trigo G., Rodríguez-Rodríguez E., Espinosa A., Pozo-Rodríguez F., Gómez F., Fuster C., Castaño-Vinyals G., Antó J.M., Barberà J.A. (2012). Persistent respiratory symptoms in clean-up workers 5 years after the Prestige oil spill. Occup. Environ. Med..

[7] Antao V.C., Pallos L.L., Graham S.L., Shim Y.K., Sapp J.H., Lewis B., Bullard S., Alper H.E., Cone J.E., Farfel M.R. (2019). 9/11 Residential Exposures: The impact of world trade center dust on respiratory outcomes of lower Manhattan residents. Int. J. Environ. Res. Public Health.

[8] Friedman S.M., Farfel M.R., Maslow C.B., Cone J.E., Brackbill R.M., Stellman S.D. (2013). Comorbid persistent lower respiratory symptoms and posttraumatic stress disorder 5–6 years post-9/11 in responders enrolled in the World Trade Center Health Registry. Am. J. Ind. Med..

[9] Friedman S.M., Farfel M.R., Maslow C., Jordan H.T., Li J., Alper H., Cone J.E., Stellman S.D., Brackbill R.M. (2016). Risk factors for and consequences of persistent lower respiratory symptoms among World Trade Center Health Registrants 10 years after the disaster. Occup. Environ. Med..

[10] Jordan H.T., Friedman S.M., Reibman J., Goldring R.M., Archie S.A.M., Ortega F., Alper H., Shao Y., Maslow C.B., Cone J.E. (2017). Risk factors for persistence of lower respiratory symptoms among community members exposed to the 2001 World Trade Center terrorist attacks. Occup. Environ. Med..

[11] Yip J., Zeig-Owens R., Hall C.B., Webber M.P., Olivieri B., Schwartz T., Kelly K.J., Prezant D.J. (2016). Health conditions as mediators of the association between World Trade Center exposure and health-related quality of life in firefighters and EMS workers. J. Occup. Environ. Med..

[12] Şakar A., Yorgancıoğlu A., Aydemir Ö., Sepit L., Çelik P. (2007). Effect of severity of asthma on quality of life. Tuberk Toraks.

[13] Bousquet J., Knani J., Dhivert H., Richard A.L.A.I.N., Chicoye A.N.N.I.E., Ware J.E., Michel F.B. (1994). Quality of life in asthma. I. Internal consistency and validity of the SF-36 questionnaire. Am. J. Respir. Crit. Care Med..

[14] Ried L.D., Nau D.P., Grainger-Rousseau T.J. (1999). Evaluation of patient’s Health-Related Quality of Life using a modified and shortened version of the Living with Asthma Questionnaire (ms-LWAQ) and the medical outcomes study, Short-Form 36 (SF-36). Qual. Life Res..

[15] Muraki M., Ichihashi H., Haraguchi R., Iwanaga T., Kubo H., Tohda Y. (2008). Comparison of the Asthma Health Questionnaire-33-Japan and the short-form 36-item health survey for measuring quality of life in Japanese patients with asthma. Allergol. Int..

[16] Brackbill R.M., Hadler J.L., Di Grande L., Ekenga C.C., Farfel M.R., Friedman S., Perlman S.E., Stellman S.D., Walker D.J., Wu D. (2009). Asthma and posttraumatic stress symptoms 5 to 6 years following exposure to the World Trade Center terrorist attack. Jama.

[17] Corporation R. (2021). 36-Item Short Form Survey (SF-36) Scoring Instructions. https://www.rand.org/health-care/surveys_tools/mos/36-item-short-form/scoring.html.

[18] Ware J.E., Sherbourne C.D. (1992). The MOS 36-item short-form health survey (SF-36). I. Conceptual framework and item selection. Med Care.

[19] Jeebhay M.F., Ngajilo D., le Moual N. (2014). Risk factors for nonwork-related adult-onset asthma and occupational asthma: A comparative review. Curr. Opin. Allergy Clin. Immunol..

[20] Soong W., Chipps B.E., O’Quinn S., Trevor J., Carr W.W., Belton L., Trudo F., Ambrose C.S. (2021). Health-Related Quality of Life and Productivity Among US Patients with Severe Asthma. J. Asthma Allergy.

[21] Song H.J., Blake K.V., Wilson D.L., Winterstein A.G., Park H. (2021). Health-related quality of life and health utilities of mild, moderate, and severe asthma: Evidence from the medical expenditure panel survey. J. Asthma Allergy.

[22] Stucky B.D., Sherbourne C.D., Edelen M.O., Eberhart N.K. (2015). Understanding asthma-specific quality of life: Moving beyond asthma symptoms and severity. Eur. Respir. J..

[23] Osman L.M., Calder C., Robertson R., Friend J.A., Legge J.S., Graham Douglas J. (2000). Symptoms, quality of life, and health service contact among young adults with mild asthma. Am. J. Respir. Crit. Care Med..

[24] de Albornoz S.C., Chen G. (2021). Relationship between health-related quality of life and subjective wellbeing in asthma. J. Psychosom. Res..

[25] Leynaert B., Neukirch C., Liard R., Bousquet J., Neukirch F. (2000). Quality of life in allergic rhinitis and asthma: A population-based study of young adults. Am. J. Respir. Crit. Care Med..

[26] Kalpaklĩoğlu A.F., Baççıoğlu A. (2008). Evaluation of quality of life: Impact of allergic rhinitis on asthma. J. Investig. Allergol. Clin. Immunol..

[27] Wijnhoven H.A., Kriegsman D.M., Snoek F.J., Hesselink A.E., De Haan M. (2003). Gender differences in health-related quality of life among asthma patients. J. Asthma.

[28] Belloch A., Perpiñá M., Martínez-Moragón E., de Diego A., Martínez-Francés M. (2003). Gender differences in health-related quality of life among patients with asthma. J. Asthma.

[29] Colombo D., Zagni E., Ferri F., Canonica G.W. (2019). Gender differences in asthma perception and its impact on quality of life: A post hoc analysis of the PROXIMA (Patient Reported Outcomes and Xolair® In the Management of Asthma) study. Allergy Asthma Clin. Immunol..

[30] Chhabra S.K., Chhabra P. (2011). Gender differences in perception of dyspnea, assessment of control, and quality of life in asthma. J. Asthma.

[31] Larsson U., Taft C., Karlsson J., Sullivan M. (2007). Gender and age differences in the relative burden of rhinitis and asthma on health-related quality of life—A Swedish population study. Respir. Med..

[32] Tovt-Korshynska M.I., Dew M.A., Chopey I.V., Spivak M.Y., Lemko I.S. (2001). Gender differences in psychological distress in adults with asthma. J. Psychosom. Res..

