# Supplementary material for: Health-Related Quality of Life (HRQoL) of Residents with Persistent Lower Respiratory Symptoms or Asthma Following a Sulphur Stockpile Fire Incident
Source: Int J Environ Res Public Health. 2022 Mar 2;19(5):2915. doi: 10.3390/ijerph19052915 (PMC8910352; doi:10.3390/ijerph19052915)
Supplement: Supplementary file 1 [file ijerph-19-02915-s001.zip › Supplementary Table S2.pdf]

Table S2. Mean difference in the health- related quality of life SF-36 scale scores of Macassar residents stratified according to the presence of persistent lower respiratory symptoms and/or asthma 6 years after the fire (*n* = 246)

Mean difference at year 1

	Role Physical		Bodily Pain		General Health	
	Mean (SD)	Median (IQR)	Mean (SD)	Median (IQR)	Mean (SD)	Median (IRR)
**PLRS/Asthma**	-36 ± 46.8	0(-100 – 0)	-16 ± 23.9	0(-22.5 – 0)	-50 ± 26.2	-56(-75 - -31)
**No PLRS/Asthma**	-24 ± 42.9	0(-25 – 0)	-14 ± 25.0	0(-22.5 – 0)	-30 ± 30.3	-25 (-56 – 0)
**p-value**	**0.036**		0.383		**<0.001**	

	Vitality		Social Functioning		Role Emotional		Mental Health	
	Mean (SD)	Median (IQR)	Mean (SD)	Median (IQR)	Mean (SD)	Median (IQR)	Mean (SD)	Median (IQR)
**PLRS/Asthma**	-21 ±21.2	-17.5 (-35 – 0)	-23 ± 24.4	- 20 (-40 – 0)	-25 ±43.2	0(-50 – 0)	-15 ± 19.4	- 12(-24 – 0
**No PLRS/Asthma**	-21 ±22.2	-15 (-35 – 0)	-18 ±24.1	-13 (-33 – 0)	-22 ± 42.7	0 (0 – 0)	-14 ± 18.3	-8 (-22 – 0)
**p-value**	0.931		0.114		0.583		0.403	

Mean difference at year 6

	Role Physical		Bodily Pain		General Health	
	Mean (SD)	Median (IQR)	Mean (SD)	Median (IQR)	Mean (SD)	Median (IRR)
**PLRS/Asthma**	-58±47.9	-100 (-100 – 0	-24 ± 27.6	-18(-5 – 0)	-50 ± 28.3	- 56 (-75 - - 25)
**No PLRS/Asthma**	-46±48.6	-25 (-100 – 0)	-24 ± 30.2	-15 (-45 – 0)	-38 ± 30.3	-37 (-63 - - 9)
**p-value**	0.098		0.983		**0.004**	

	Vitality	Social Functioning	Role Emotional	Mental Health

	Mean (SD)	Median (IQR)	Mean (SD)	Median (IQR)	Mean (SD)	Median (IQR)	Mean (SD)	Median (IQR)
**PLRS/Asthma**	-31 ± 23.3	-30(-50 - -15)	-31 ± 28.0	- 28 (-58 – 0	-47 ± 49.3	0 (-100 – 0)	-23 ± 19.1	-16 (-40 – 0
**No PLRS/Asthma**	-29 ±23.1	-30 (-50 - -10)	-27 ± 28.3	- 23 (-45 – 0)	- 47 ± 49.2	0 (-100 – 0)	-19±.19.5	-12(-34-0)
**p-value**	0.431		0.276		0.812		0.768	

Mann-Whitney test
