# Supplementary material for: Health-Related Quality of Life (HRQoL) of Residents with Persistent Lower Respiratory Symptoms or Asthma Following a Sulphur Stockpile Fire Incident
Source: Int J Environ Res Public Health. 2022 Mar 2;19(5):2915. doi: 10.3390/ijerph19052915 (PMC8910352; doi:10.3390/ijerph19052915)
Supplement: Supplementary file 1 [file ijerph-19-02915-s001.zip › Supplementary Tables S1.pdf]

Table S1. Health- related quality of life SF-36 scale scores of Macassar residents prior to and at 1 year and 6 years after the fire (*n* = 246).

	Mean ± SD	Median (IQR)	p-value
**Role Physical**			
Prior to fire	94 ± 23.0	100 (100-100)	
Year 1	66 ± 46.3	100 (0-100)	**<0.001**
Year 6	44 ± 48.3	0 (0-100)	
**Bodily Pain**			
Prior to fire	92 ± 15.2	100 (78-100)	
Year 1	76 ± 26.7	78 (55-100)	**<0.001**
Year 6	64 ± 30.9	78 (33-100)	
**General Health**			
Prior to fire	75 ± 18.1	81 (69-88)	
Year 1	39 ± 25.3	31(19-56)	**<0.001**
Year 6	33 ± 24.8	25(13-50)	
**Vitality**			
Prior to fire	82 ± 18.1	85 (75-100)	
Year 1	61 ± 22.9	60 (45-80)	**<0.001**
Year 6	51 ± 20.6	50 (40-65)	
**Social Functioning**			
Prior to fire	88 ± 16.3	100 (80-100)	
Year 1	69 ± 25.5	68 (48-80)	**<0.001**
Year 6	60 ± 28.6	65 (43-80)	
**Role Emotional**			
Prior to fire	95± 21.5	100 (100-100)	
Year 1	72 ± 44.6	100 (0-100)	**<0.001**
Year 6	49 ± 49.3	50 (0-100)	
**Mental Health**			
Prior to fire	82± 19.0	85 (75-100)	
Year 1	61± 22.9	60 (45-80)	**<0.001**
Year 6	51 ± 20.6	50 (40-65)	
